# Dietary Anthocyanins against Obesity and Inflammation

**DOI:** 10.3390/nu9101089

**Published:** 2017-10-01

**Authors:** Yoon-Mi Lee, Young Yoon, Haelim Yoon, Hyun-Min Park, Sooji Song, Kyung-Jin Yeum

**Affiliations:** 1 Division of Food Bioscience, College of Biomedical and Health Sciences, Konkuk University, Chungju-si 27478, Korea; yoonmilee@kku.ac.kr (Y.-M.L.); lab_yyoung0418@naver.com (Y.Y.); limtiny@naver.com (H.Y.); loveangela0312@gmail.com (H.-M.P.); ssj4037@naver.com (S.S.); 2 Nanotechnology Research Center, Konkuk University, Chungju-si 27478, Korea; 3 Institute of Biomedical and Health Science, Konkuk University, Chungju-si 27478, Korea

**Keywords:** obesity, inflammation, anthocyanin, flavonoids

## Abstract

Chronic low-grade inflammation plays a pivotal role in the pathogenesis of obesity, due to its associated chronic diseases such as type II diabetes, cardiovascular diseases, pulmonary diseases and cancer. Thus, targeting inflammation is an attractive strategy to counter the burden of obesity-induced health problems. Recently, food-derived bioactive compounds have been spotlighted as a regulator against various chronic diseases due to their low toxicity, as opposed to drugs that induce severe side effects. Here we describe the beneficial effects of dietary anthocyanins on obesity-induced metabolic disorders and inflammation. Red cabbage microgreen, blueberry, blackcurrant, mulberry, cherry, black elderberry, black soybean, chokeberry and jaboticaba peel contain a variety of anthocyanins including cyanidins, delphinidins, malvidins, pelargonidins, peonidins and petunidins, and have been reported to alter both metabolic markers and inflammatory markers in cells, animals, and humans. This review discusses the interplay between inflammation and obesity, and their subsequent regulation via the use of dietary anthocyanins, suggesting an alternative dietary strategy to ameliorate obesity and obesity associated chronic diseases.

## 1. Introduction

Obesity is accompanied by metabolic disturbances such as dyslipidemia, hyperglycemia and hypertension. The burden of obesity is largely derived from its associated chronic diseases such as cardiovascular diseases, pulmonary diseases, cancer, and type II diabetes mellitus [1,2]. Continuous effort has been made to understand the pathogenesis between obesity and chronic diseases. Interestingly, chronic low-grade inflammation has been linked to the progression of obesity and its related diseases [3,4].

Inflammation is a defense system that responds to harmful stimuli and restores disruptive tissues back to homeostasis [5]. Upon recognizing unfavorable stimuli, including excessive nutrients, immune cells urgently secrete a number of cytokines and chemokines to the damaged area, defined as innate immunity. If the condition is prolonged, antigen-presenting cells (APC) and B- and T lymphocytes perform adaptive immunity which may increase the prevalence of obesity-associated chronic diseases [6]. Thus, preventing this prolonged pro-inflammatory condition can be a valid strategy against obesity-associated metabolic disorders [7,8].

Although there are a number of drugs that are approved for the treatment of obese patients, many of them were withdrawn due to the severe adverse events such as heart diseases and psychiatric disorders [9]. Meanwhile, recent studies demonstrated that consumption of food-derived bioactive components such as phenolic compounds is positively associated with reducing the risk of obesity and its associated chronic diseases with low toxicity [10,11]. Thus, creating new dietary treatments based on various bioactive components in food has been emerging as a new possible intervention against obesity [12,13].

**Figure 1:**
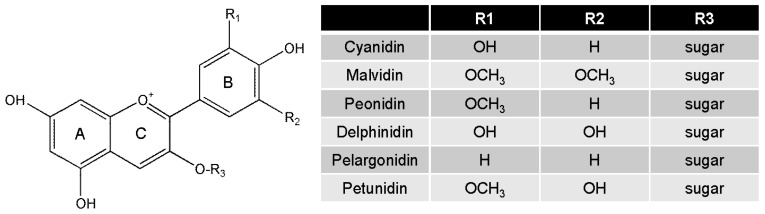
Structure of the most common anthocyanins.

Anthocyanins are an important subfamily of flavonoids, which are abundant in flowers, fruits, seeds and plant leaves [14]. The basic structural form 2-phenylchromenylium is further classified into six major compounds: cyanidin, delphinidin, malvidin, pelargonidin, peonidin and petunidin, depending on the flavylium B-ring. Sugars (glucose, arabinose, galactose, etc.) can be attached to the main structure of anthocyanins [12,15] (Figure 1). Because anthocyanins are commonly consumed, their biological activities have been extensively studied. Notably, anthocyanins have antimicrobial, antioxidative, anti-inflammatory, and anti-mutagenic properties, which in turn play a role on the prevention and treatment of many chronic diseases such as metabolic disorders, cancer, eye diseases and cardiovascular diseases [12,16,17]. In particular, mixtures of anthocyanins found in food rather than their individual anthocyanin components has been reported to be more beneficial for improving human health [18].

Collectively, over the last two decades, the link between obesity and inflammation has been highlighted as a new axis to be targeted for intervention. Dietary bioactive components that have beneficial effects against both obesity and inflammation can potentially be a modulator of this axis. Here we discuss the impact of dietary anthocyanins on alleviating obesity and inflammation.

## 2. Metainflammation and Its Mechanism

Obesity is accompanied by a chronic low-grade inflammatory condition known as metainflammation [19,20]. Pro-inflammatory markers such as interleukin-6 (IL-6), interleukin-1β (IL-1β), C-reactive protein (CRP) and tumor necrosis factor-α (TNF-α) were reported to be persistently expressed in adipocytes of white adipose tissue [19]. It is interesting to note that pro-inflammatory markers were significantly higher in obese patients compared to healthy individuals. Furthermore, obese patients with high IL-6 levels also had increased CRP levels, which is a critical risk factor for cardiovascular diseases and type 2 diabetes [13,21]. In addition, M1 macrophages, T cells, B cells and monocyte chemoattractant protein-1 (MCP-1) were also increased in adipose tissue, whereas there was a reduction in the number of regulatory T cells, M2 macrophages and amount of adiponectin [22]. The possible underlying mechanism is that toll-like receptors (TLRs), in particular TLR4, recognize external stimuli including over-nutrition, and connect them to transcription factors such as nuclear factor Kappa B (NF-κB), activated protein-1 (AP-1) and interferon regulatory factor 3 (IRF3). These inflammation-associated transcription factors enter into the nucleus and bind to the site of target genes that promote inflammation [23]. Due to the coordination between the immune system and metabolism, developing novel immunotherapeutic strategies to simultaneously attenuate obesity-related metabolic disorders seems promising. Indeed, targeting IL-1β, IL-1β receptor, TNF-α, TLRs, T and B lymphocytes were effective in reducing blood glucose levels, insulin resistance and other metabolic markers in obesity [7,8]. Flavonoids have also been assessed to regulate inflammation and obesity by lowering IL-1α, and IFN-γ compared to control mice [24]. These efforts have diversely been attempted using cell, animal and human models [25].

## 3. Establishment of an Obesity Model 

Cells and animals are essential surrogates for human obesity due to the limitations of promising human studies. In this section, we introduce frequently used cell and animal models of human obesity (Table 1).

Fat accumulation in adipose tissue is critical for obesity. Adipose tissue is composed of adipocytes, macrophages, mesenchymal stem cells, blood cells, fibroblasts, pericytes, and smooth muscle cells [26]. Cells are important in order to identify the mechanism of key adipogenic process. Mouse cell lines to study adipogenesis can be classified into three categories: mesenchymal stem cells, embryonic cells, and primary preadipocytes [27]. Among them, 3T3-L1 is the most frequently used cell line, due to its well-established methodology for mature adipocytes. When adipogenic stimuli such as insulin (1 to 10 μg/mL), dexamethasone, (1 μM) and 3-isobutyl-1-methylxanthine (IBMX) (0.5 mM) are added to medium containing 10% fetal bovine serum, several transcriptional factors such as peroxisome proliferator-activated receptor gamma (PPAR-γ), CCAAT/enhancer-binding proteins (C/EBPs) and sterol regulatory element binding (SREBP) promote adipocyte fate, resulting in fat accumulation in mature adipocyte within 14 days [28]. Mouse cell models can be easily applied to study adipogenesis in obesity, but there are some metabolic differences with human adipocytes [29]. Therefore, human preadipocyte and adipose-derived stem cells were developed, which also require adipogenic stimuli (Insulin, dexamethasone, IBMX, biotin, rosiglitazone) for differentiation to mature adipocytes by transcriptional cascades within 12–14 days [30]. Recent studies of obesity in vitro have utilized not only the above described cell models but also utilized co-culture and three dimensional culture systems [31].

Obese animals can be categorized into two groups, monogenic and polygenic models [32]. In monogenic models, ob/ob mice are C57BL/6 that spontaneously become obese due to a mutation in the leptin gene responsible for controlling appetite [33,34]. Zucker rats are a monogenic obese model where the rats are autosomal recessive for a mutation in leptin receptors, and consequently exhibit metabolic symptoms such as hyperglycemia and insulin resistance [35]. Despite the metabolic disorder phenotype displayed in these monogenic models, these models are limited in their representation of human obesity since they are induced via a mutation of a gene [36]. Diet-induced obesity models have been alternatively suggested to overcome this limitation. These animals are instead fed with high energy diets such as high fat (45% or 60% kcal energy from fat as per difference of lard contents), high-fructose and high-cholesterol diets. C57BL/6 and A/J mice are commonly used [37]. Wistar, Sprague-Dawley, Long Evans and Osborne mendel rat species are commonly used as high energy diet-induced obese rat model [38]. These polygenic models developed by high energy diets have shown the characteristic symptoms of metabolic disorders such as glucose intolerance and upregulation of cholesterol and triglycerides in the plasma of animals [32]. Diet-induced obese rats are another polygenic model where rats have been bred selectively through several generations to become obese without high energy diet supplementation [39]. These valid models may lead us to predict the mechanism and efficacy of target molecules for human obesity.

## 4. Biological Functions of Anthocyanins

Anthocyanins are well-known antioxidants that eliminate reactive oxygen species (ROS). It has been proven that anthocyanins, in particular cyanidin-3-glucoside, has great oxygen radical absorbance capacity (ORAC) in vitro [40]. Delphinidin has been revealed as the most active scavenger against superoxide anion [41]. Furthermore various reports have shown that anthocyanin has protective effects against oxidative stress in cell lines [42,43,44]. Several studies have also demonstrated the antioxidant functions of anthocyanins in vivo. Cyanidin-3-glucoside improved oxidative stress-induced hepatic ischemia-reperfusion in rats [45]. Cyanidin, delphinidin, and malvidin induced antioxidant enzymes have been reported to induce upregulation of antioxidant response element (ARE) pathways [46].

Numerous studies have demonstrated the anti-inflammatory effects of anthocyanins as well. Cyanidin-3-glucoside, delphinidin-3-glucoside and petunidin-3-glucoside inhibited NF-κB activities via mitogen activated protein kinase (MAPK) pathways [47,48,49], and cyanidins inhibited cyclooxygenase enzyme activities [50]. Additionally, cyanidin-3-glucoside had effects on reducing lung inflammation in rats [51].

These biological activities of anthocyanins are closely related to the incidence of chronic diseases. Indeed, anthocyanins reduced risks factors for cardiovascular diseases [17] and suppressed cell growth in various cancer cell lines, indicating that anthocyanins have anticancer properties [52]. Consumption of anthocyanins reduced body weight and insulin resistance, leading to restored glucose tolerance [53,54].

## 5. Bioavailability of Anthocyanins

Most bioactive compounds which are well-known for their great health benefits are generally ingested by food, the study of food as a mixture of its bioactive compounds is more relevant. Supplementation of anthocyanin mixtures reduced inflammatory markers in hypercholesterol subjects rather than individual anthocyanins [18]. Combinations of blackberries and raspberries, for instance, have synergistic antioxidant capacity [55].

Besides the total content of bioactive compounds in food, bioavailability including metabolism and excretion is critical as well. Although anthocyanins have been reported to have low bioavailability after evaluating plasma levels after ingestion of anthocyanin-rich food, recent studies have started to focus on anthocyanin metabolites which were not able to be detected previously, using traditional methods. Interestingly, bioavailability of anthocyanin metabolites was reported to be higher than that of parent anthocyanins by 42-fold [56]. Newly identified anthocyanin metabolites contribute to the improvement of human health [57].

Furthermore, various attempts have been made to improve the bioavailability of flavonoids including anthocyanins using a novel delivery system. Nanoparticles, encapsulation of food, liposomes and gel emersions have been studied to enhance intestinal absorption of food [58]. Encapsulated anthocyanins in liposomes and alginate/chitosan microencapsulation of anthocyanins were valuable for increasing bioefficacy, suggesting application of anthocyanins on nutraceutical development [59,60].

## 6. Studies of Dietary Anthocyanins on Regulation of Inflammation and Obesity in Various Models

Table 2 presents the beneficial effects of dietary anthocyanins on obesity-related metabolic markers and inflammatory markers in cells and animals. Because there are many studies with respect to the effects of anthocyanins on obesity and inflammation, we look at studies of dietary anthocyanins which are clearly defined for their bioactive compounds on metabolism and inflammation in specific models.

It is difficult to demonstrate anti-inflammatory and anti-adipogenic effects in the same cell line due to each cell’s different characteristics. One study reported that purple sweet potato extract exerted antilipogenic activities by suppression of adipogenic enzymes and transcription factors, and had an anti-inflammatory effect by down-regulating COX-2, IL-6 and MCP-1 in 3T3-L1 mouse adipocytes [61].

Red cabbage microgreen decreased weight gain, low density lipoprotein (LDL) levels, triacylglycerol, and cholesterol levels in high fat diet-fed mice. Inflammatory cytokines such as CRP and TNF-α were also significantly diminished in mice [62]. Since blueberries contain a distinct amount of anthocyanins, this has attracted several research studies on their health benefits. High fat diets containing blueberry-fed mice have shown reduced body weight and blood glucose levels as well as TNF-α and IL-6 levels than that of high fat diet-fed control mice [63]. In addition, another study demonstrated that whole blueberry improved high fat diet-induced insulin resistance, and decreased TNF-α, IL-6, MCP-1, CD11c+, and inducible nitric oxide synthase (iNOS) [64]. Blueberry juice was also effective in reducing body weight, insulin and leptin levels in serum, cholesterol and triacylglycerol in the liver, and inflammatory markers such as TNF-α, IL-6, iNOS, and NF-κB were decreased in the epididymal adipose tissue of high fat diet-fed mice. This efficacy was also similarly observed in mulberry juice-supplemented obese mice [65]. Other anthocyanin-rich blackcurrant [66], mulberry, cherry [67], black elderberry [68], black soybean [69], freeze-dried jaboticaba peel [70] lowered fat accumulation, triacylglycerol, cholesterol levels in blood serum or liver in high fat diet-fed mice. Rats ingesting fructose rich diets appeared to become obese. However, chokeberry extract was added to the drinking water of the rats, leading to improved metabolic disturbance and inflammation [71]. In addition, tart cherry reduced metabolic and inflammatory factors in Zucker fatty rats [72].

Several clinical studies have also been conducted to evaluate biological functions of dietary anthocyanins on inflammation and obesity, as shown in Table 3. Due to the lack of clinical studies, studies without characterization of anthocyanins were also included in the list. Human subjects whose BMI (body mass index) is over 23 or have a waist circumference over 90 cm (over 85 cm in case of female) were allocated to either placebo or black soybean extract-administered groups. This randomized, double-blinded, clinical study showed reduction in abdominal fat, cholesterol, triacylglycerol and LDL levels, with decreasing TNF-α and MCP-1 levels [73]. Red orange juice was also effective in reducing metabolic markers and inflammatory markers in human subjects.

## 7. Summary

Previously, obesity was regarded as just a matter of excessive energy storage triggered by energy imbalance, however many studies have indicated that chronic low-grade inflammation in adipose tissue can be an important issue in obesity. In particular, this metainflammation could enable pathogenesis of chronic diseases associated with obesity. Anthocyanins have great biological activities and low toxicity in vivo, therefore many scientists are interested in the health benefits of anthocyanins, as well as their application in preventing and treating chronic diseases, including obesity. Here, we describe the positive effects of dietary anthocyanins limited to their well-defined components against metabolic and inflammatory markers in cell, animal and human obesity models. In addition, anthocyanin mixtures found in food such as red cabbage microgreen, blueberry, blackcurrant, mulberry, cherry, black elderberry, black soybean, chokeberry and jaboticaba peel (in whole or extract) interestingly had higher clinical efficacy than single anthocyanins.

## 8. Conclusions

**Figure 2:**
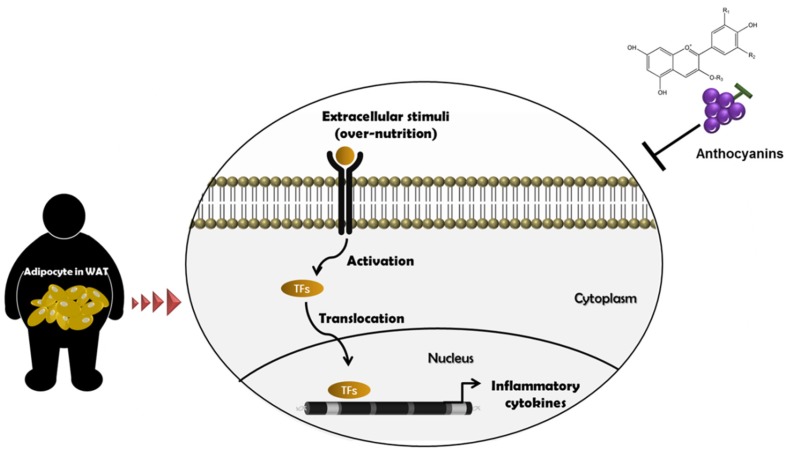
Beneficial effects of anthocyanins on obesity and inflammation. When receptors recognize the status of over-nutrition, they activate various transcription factors such as NF-κB, IRF-3 and AP-1 for translocation into nucleus and bind to the promoter region of target genes. Eventually, inflammatory cytokines are expressed resulting in chronic inflammatory conditions in adipocytes of WAT. Dietary anthocyanins may have preventive effects on these events, leading to health benefits.

We reviewed the effects of anthocyanins-rich food on attenuating obesity and inflammation in cells, animals, and humans. Taken together, dietary anthocyanins may be a potential regulator of obesity-derived inflammation and its associated chronic diseases, as presented in Figure 2.

## Figures and Tables

**Table 1:** Frequently used obesity model in vitro and in vivo.

			Advantage	Disadvantage
In vitro	mouse	mesenchymal stem cells embryonic stem cells primary preadipocytes	easy to study mechanism, well-established method	different characteristics of human vs. mouse
	human	preadipocytes adipose-derived stem cells	easy to study mechanism, representation of human obesity	complement of mouse cell lines‘ disadvantages
In vivo	monogenic	ob/ob mice Zucker fatty rats	apparent symptoms of metabolic disorders	monogenic mutation is rare in humans
	polygenic	High energy diets-fed mice Diet-induced obesity	resemble complexities of human obesity	complement of monogenic models‘ disadvantages

**Table 2:** Effects of dietary anthocyanins on obesity and inflammation (cell and animal study).

Food Sources	Identified Bioactive Dose of Anthocyanins	Mediators	Inducer	Metabolic Marker	Inflammatory Marker	Ref.
Purple sweet potato	cyanidnin	3T3-L1	Stimuli vs. undifferentiated cells			[61]
	(3-caffeylferulysophoroside-5-glucoside)			leptin ↓	COX-2 ↓	
	peonidin			adipogenic factors ↓	MCP-1 ↓	
	(3-caffeylferulysophoroside-5-glucoside)				IL-6 ↓	
	Dose: 4.28 μg/mL to 12.84 μg/mL ^a^					
Red cabbage microgreen	cyanidin	mice (C57BL/6)	high fat-diet vs. normal-diet for 8 weeks			[62]
	(3-diglucoside-5-glucoside)					
	cyanidin					
	(3-(sinapoyl)-diglucoside-5-glucosides)					
	cyanidin					
	(3-(glucosyl)(sinapoyl)(p-coumaroyl)sophorside-5-glucoside)					
	cyanidin					
	(3-(glucosyl)(sinapoyl)(feruloyl)sophorside-5-glucoside)			LDL ↓	CRP ↓	
	cyanidin			cholesterol ↓	TNF-α ↓	
	(3-diferuloylsophoroside-5-glucoside)			TG ↓		
	cyanidin					
	(3-(coumaroyl)sophoroside-5-glucoside)					
	cyanidin					
	(3-(feruloyl)sophoroside-5-glucoside)					
	cyanidin					
	(3-diferuloylsophoroside-5-glucoside)					
	cyanidin					
	(3-(sinapoyl)(feruloyl)sophoroside-5-glucoside)					
	cyanidin					
	(3-(sinapoyl)(sinapoyl)sophoroside-5-glucoside)					
	Dose: 139.596 nmol/g ^b^					
Blueberry	delphinidins	mice (C57BL/6)	high fat-diet vs. normal diet for 8 weeks	glucose ↓	TNF-α ↓ IL-6 ↓ MCP-1 ↓ iNOS ↓ IL-10 ↑ CD11c+	[64]
	cyanidins					
	peonidins					
	malvidins					
	Dose: 1.29 mg/g ^b^					
Blueberry	cyanidin	mice (C57BL/6)	high fat-diet vs. normal diet for 8 weeks			[63]
	(3-galactoside)					
	cyanidin					
	(3-arabinoside)					
	delphinidin					
	(3-arabinoside)			glucose ↓	TNF-α ↓	
	delphinidin			TG ↓	IL-6 ↓	
	(3-galactoside)			cholesterol ↓		
	petunidin			insulin ↓		
	(3-glucoside)			leptin ↓		
	petunidin					
	(3-arabinoside)					
	malvidin					
	(3-galactoside)					
	malvidin					
	(3-glucoside)					
	Dose: 50 to 200 μg/g ^b^					
Black elderberry	cyanidin	mice (C57BL/6)	high fat-diet vs. normal diet for 16 weeks			[68]
	(3-glucoside)			TG ↓	MCP-1 ↓	
	cyanidin			insulin ↓	TNF-α ↓	
	(3-sambubioside)			cholesterol ↓		
	Dose: 3.334, 1.7 μg/g ^b^					
Blackcurrant	delphinidin	mice (C57BL/6)	high fat/cholesterol-diet vs. normal diet for 12 weeks	adipogenic genes ↓		[66]
	(3-glucoside)					
	delphinidin					
	(3-o-rutinoside)				TNF-α ↓	
	cyanidin				IL-6 ↓	
	(3-glucoside)				IL-1β ↓	
	cyanidin					
	(3-rutinoside)					
	Dose: 298.1 μg/g ^b^					
Mulberry	cyanidin	mice (C57BL/6)	high fat-diet vs. normal diet for 16 weeks			[67]
	(3-glucoside)				TNF-α ↓	
	cyanidin			glucose ↓	IL-6 ↓	
	(3-rutinoside)			leptin ↓	iNOS ↓	
	pelarginidin				NF-κB ↓	
	(3-glucose)					
	Dose: 200 μg/g ^b^					
Cherry	cyanidin	mice (C57BL/6)	high fat-diet vs. normal diet for 16 weeks			[67]
	(3-2G-glucosylrutinoside)					
	cyanidin				TNF-α ↓	
	(3-rutinoside)			glucose ↓	IL-6 ↓	
	pelarginidin			leptin ↓	iNOS ↓	
	(3-rutinoside)				NF-κB ↓	
	Dose: 200 μg/g ^b^					
Blueberry juice	cyanidin	mice (C57BL/6)	high fat-diet vs. normal diet for 12 weeks			[65]
	(3-galactoside)					
	cyanidin					
	(3-arabinoside)					
	delphinidin					
	(3-glucoside)					
	delphinidin			leptin ↓		
	(3-galactoside)			cholesterol ↓	TNF-α ↓	
	delphinidin			adiponectin ↑	IL-6 ↓	
	(3-arabinoside)			TG ↓		
	petunidin					
	(3-glucoside)					
	petunidin					
	(3-arabinoside)					
	malvidin					
	(3-galactoside)					
	malvidin					
	(3-glucoside)					
	Dose: 4.09 mg/mL ^c^					
Mulberry juice	cyanidin	mice (C57BL/6)	high fat-diet vs. normal diet for 12 weeks			[65]
	(3-glucoside)					
	cyanidin					
	(3-rutinoside)			leptin ↓	TNF-α ↓	
	pelargonidin			adiponectin ↑	IL-6 ↓	
	(3-glucoside)					
	pelargonidin					
	(3-rutinoside)					
	Dose: 21.86 mg/mL ^c^					
Black soybean	delphinidin	mice (C57BL/6)	high fat-diet vs. normal diet for 12 weeks			[69]
	(3-glucoside)					
	cyanidin					
	(3-glucoside)					
	petunidin			TG ↓	TNF-α ↓	
	(3-glucoside)			cholesterol ↓	IL-6 ↓	
	pelargonodin				IL-10↑	
	(3-glucoside)					
	peonidin					
	(3-glucoside)					
	Dose: 12.48 mg/g ^b^					
Jaboticaba peel	delphinidin	swiss inbred mice	high fat-diet vs. normal diet for 6 weeks	insulin ↓		[70]
	(3- *O*-glycoside)				IL-6 ↓	
	cyanidin				IL-1β ↓	
	(3- *O*-glycoside)					
	Dose: 259.9, 519.8, 1039.6 μg/g ^b^					
Chokeberry	total anthocyanin	Wistar rat	fructose-rich diet vs. normal diet for 6 weeks	glucose ↓ insulin ↓ TG ↓ cholesterol ↓	TNF-α ↓	[71]
	Dose: 10 or 20 mg/kg ^d^				IL-6 ↓	
Tart cherry	cyanidin	zucker fatty rats	Spontaneously obese for 90 days			[72]
	(3-sophoroside)					
	cyanidin					
	(3-glucosylrutinoside)					
	cyanidin-			glucose ↓		
	(3-glucoside)			insulin ↓	TNF-α ↓	
	cyanidin			cholesterol ↓	IL-6 ↓	
	(3-rutinoside)			TG ↓		
	peonidin					
	(3-glucoside)					
	Pelargonidin					
	Dose: 0.6598 mg/g ^b^					

^a^: cyanidin concentration of extract, ^b^: the amount of anthocyanidins per weight of diet, ^c^: the amount of anthocyanidins per volume of diet, ^d^: the amount of anthocyanidin per body weight. CRP: C-reactive protein.

**Table 3:** Effects of dietary anthocyanins on obesity and inflammation (clinical study).

Food Sources	Bioactives Dose of Anthocyanins	Subject Duration	Metabolic Marker	Inflammatory Marker	Ref.
Black soybean	cyanidin				[73]
	(3-glucosides)	BMI > 23			
	delphinidin	WC > 90 for male	TG ↓	TNF-α ↓	
	(3-glucoside)	WC > 85 for female	cholesterol ↓	MCP-1 ↓	
	petunidin	For 8 weeks	LDL ↓		
	(3-glucoside)				
	31.48 mg/day				
Red orange juice	Anthocyanin mixture 250 mg/day	average BMI = 34.4 ± 4.8 for 12 weeks	Δ leptin ↓	CRP ↓	[74]
			Δ adiponectin ↓	TNF-α ↓	
Red-fleshed sweet orange juice	anthocyanin mixture 750 mL/day	age 23–59 BMI 18.5–24.986.4/74.6	cholesterol ↓	CRP↓	[75]

BMI: body mass index; WC: waist circumstance; CRP: C-reactive protein; Δ: incremental change.

## Abbreviation

TG	triacylglycerol
LDL	low density lipoprotein
COX-2	cyclooxygenase-2
TNF-α	tumor necrosis factor-α
IL-6	interleukin-6
IL-1β	interleukin-1β
IL-10	interleukin-10
iNOS	inducible nitric synthase
MCP-1	monocyte chemoattactant protein-1
NF-κB	nuclear factor kappa B
BMI	body mass index
CRP	c-reactive protein
WC	waist circumstance
IRF-3	interferon regulatory factor 3
AP-1	activated protein-1
WAT	white adipose tissue
